# Jmol SMILES and Jmol SMARTS: specifications and applications

**DOI:** 10.1186/s13321-016-0160-4

**Published:** 2016-09-26

**Authors:** Robert M. Hanson

**Affiliations:** Department of Chemistry, St. Olaf College, 1520 St. Olaf Ave., Northfield, MN USA

## Abstract

**Background:**

SMILES and SMARTS are two well-defined structure matching languages that have gained wide use in cheminformatics. Jmol is a widely used open-source molecular visualization and analysis tool written in Java and implemented in both Java and JavaScript. Over the past 10 years, from 2007 to 2016, work on Jmol has included the development of dialects of SMILES and SMARTS that incorporate novel aspects that allow new and powerful applications.

**Results:**

The specifications of “Jmol SMILES” and “Jmol SMARTS” are described. The dialects most closely resemble OpenSMILES and OpenSMARTS. Jmol SMILES is a superset of OpenSMILES, allowing a freer format, including whitespace and comments, the addition of “processing directives” that modify the meaning of certain aspects of SMILES processing such as aromaticity and stereochemistry, a more extensive treatment of stereochemistry, and several minor additions. Jmol SMARTS similarly adds these same modifications to OpenSMARTS, but also adds a number of additional “primitives” and elements of syntax tuned to matching 3D molecular structures and selecting their atoms. The result is an expansion of the capabilities of SMILES and SMARTS primarily for use in 3D molecular analysis, allowing a broader range of matching involving any combination of 3D molecular structures, SMILES strings, and SMARTS patterns. While developed specifically for Jmol, these dialects of SMILES and SMARTS are independent of the Jmol application itself.

**Conclusions:**

Jmol SMILES and Jmol SMARTS add value to standard SMILES and SMARTS. Together they have proven exceptionally capable in extracting valuable information from 3D structural models, as demonstrated in Jmol. Capabilities in Jmol enabled by Jmol SMILES and Jmol SMARTS include efficient MMFF94 atom typing, conformational identification, SMILES comparisons without canonicalization, identification of stereochemical relationships, quantitative comparison of 3D structures from different sources (including differences in Kekulization), conformational flexible fitting, and atom mapping used to synchronize interactive displays of 2D structures, 3D structures, and spectral correlations, where data are being drawn from multiple sources.

**Electronic supplementary material:**

The online version of this article (doi:10.1186/s13321-016-0160-4) contains supplementary material, which is available to authorized users.

## Background

The Simplified Molecular-Input Line-Entry System (SMILES) [1–3] and SMILES Arbitrary Target Specification (SMARTS) [4, 5] have been of tremendous value in the area of cheminformatics. Developed in the late 1980s, these languages have found wide application, particularly in relation to small primarily organic molecules. In addition, SMILES has been extended in the form of CHUCKLES [6] and CHORTLES [7] (an extension of CHUCKLES), both for biopolymers, and CurlySMILES (an annotated version of SMILES) [8]. Alternatives to SMARTS-based molecular querying include Sybyl Line Notation (SLN) [9, 10], which itself is an adaption of SMILES, the relatively underdeveloped Molecular Query Language (MQL) [11], and the XML-based Chemical Subgraphs and Reactions Markup Language (CSRML) [12]. And certainly programs such as Jmol [13], PyMOL [14], VMD [15], and Chimera [16] all have some sort of native selection language. Some of these languages have very powerful methods of matching molecular structures or substructures with query criteria.

This article focuses on the development of SMILES and SMARTS dialects that can be used specifically in the context of a 3D molecular visualization environment to answer not only the typical questions such as whether two structures and/or SMILES strings match or whether a particular 3D structure and/or SMILES string contain some particular 3D substructure (practical examples 1 and 2, below), but also more challenging questions (practical examples 3–8, below) such as:

- Given two 3D structures, what is their isomeric relationship?
- Given two 3D structures from two different sources, how quantitatively similar are they?
- How can I align two 3D models in order to visualize their similarity?
- What would I need to do to the given conformation of Structure A to match it conformationally with Structure B? or with some substructure within B?
- Given a 3D structure, what is its conformation? For example, if it is a cyclohexane, is it in the chair or boat form? Are substituents axial or equatorial?
- How can I correlate 2D and 3D chemical structures from different sources? For example, how can I correlate a given 2D or 3D structure with a simulated NMR spectrum?

In this article I introduce adaptations to SMILES and SMARTS that address all of these questions, allowing them to be answered immediately and definitively. In the case of on-line browser-based applications, these answers can be obtained completely within the standard browser client, without access to external dedicated cheminformatics services. While the development of Jmol SMILES and Jmol SMARTS was—not surprisingly—Jmol, it is important to emphasize that nothing that is presented here is limited to use in Jmol. All of the additions to SMILES and SMARTS presented are simple and straightforward. The success of implementing Jmol SMILES and Jmol SMARTS within Jmol simply provides an example of the continued power of SMILES and SMARTS in the cheminformatics open-source community.

## Implementation

The context for this work is Jmol, a widely used open-source community-driven program for the visualization and analysis of molecular structure [12]. Jmol has been used in a broad range of contexts, including small organic and inorganic molecules, biomolecules, and crystallographic structures crossing the boundaries of biology, chemistry, physics, and materials science. The Jmol application is written in Java and implemented (in parallel) in both Java and JavaScript. It is available in three formats: as a stand-alone desk-top or batch-driven Java program, a Java applet, and an HTML5 JavaScript-only equivalent (JSmol). The reference implementation for this article is Jmol 14.6.1_2016.07.11.

The dialects of SMILES and SMARTS implemented here are referred to as “Jmol SMILES” and “Jmol SMARTS” respectively, but there is nothing specific to Jmol in those descriptions. As such, Jmol SMILES and Jmol SMARTS could be implemented if desired in any 3D molecular visualization platform, such as PyMOL, VMD, or Chimera. Jmol SMILES most closely resembles OpenSMILES [3], while Jmol SMARTS is based on OpenSMARTS [5]. Jmol SMILES is a superset of OpenSMILES, allowing a freer format, with optional comments and whitespace, optional “processing directives” that specify the meaning of certain aspects of SMILES processing such as aromaticity, a more complete treatment of stereochemistry, and several other minor additions. Jmol SMARTS similarly adds these same modifications to OpenSMARTS, as well as several additional “primitives” and elements of syntax specifically tuned to the investigation of 3D structural models.

To keep this in perspective, imagine that we have before us a *single* molecular structure. Perhaps it is a structure loaded into JSmol on a web page, perhaps from a student drawing a 2D structure with an editor. The developer of the page may not have any a priori information about what structure is present. Did the student draw a ketone (as was requested, perhaps)? Did they properly identify the diene and dienophile in a Diels–Alder reaction? These are the sorts of questions that Jmol is capable of investigating, and for which SMILES and SMARTS matching can be extremely valuable. In addition, we will see that the real power in the use of SMILES and SMARTS in a program such as Jmol can be *behind the scenes*, totally hidden from the user, powering the functionality that to the user appears simple, nearly instantaneous, and possibly almost magical.

To understand the significance behind the development of Jmol SMILES and Jmol SMARTS (as opposed to just using standard versions of such), it is important to understand a little about how Jmol works. When loading chemical structures, Jmol creates a linear array of N atoms starting with index 0 and going through index N − 1. These atoms may all represent one model, where a “model” could be a single protein structure, or an organic molecule, or a crystal structure. Thus, a “model” in Jmol is a sequential set of atoms. When there are multiple models, they might be from a single source (an external database or a locally saved structure), or they may be from different sources (one from PubChem [17], the other from NCI/CADD [18]); they may be multiple models from the loading of a single file or several files; one might be drawn by a student using a web-based 2D drawing app; the other a 3D reference the student may or may not have access to). Whatever the case, we are interested in answering questions that correlate the given 3D representation of the model with one or more other representations—perhaps a SMILES string, a SMARTS pattern, a 2D structural model, or another 3D model.

While this paper is not meant to be a Jmol tutorial, some explanation of the Jmol examples is in order. Notation such as {2.1} in the tables and discussion below refers to a model—in this case, “the atoms associated with the first model in the second file loaded.” Notation ({0:24}) refers to the first 25 atoms in Jmol’s atom array. ({0 5}) refers to two selected atoms. Words in CAPITALS such as **LOAD**, **SELECT**, **PRINT**, and **SHOW**, are Jmol command tokens; words in lower case followed by parentheses, such as search(…), smiles(…), compare(…), and find(…) are Jmol functions. This capitalization is just a convention for this paper; capitalization in Jmol for commands tokens, variable names, and function name is not significant. So **SELECT {2.1}** selects all atoms in the first model of the second file loaded, as does **select {2.1}**. Functions smiles(…) and search(…) are Jmol functions specifically requesting SMILES and SMARTS searches, respectively. For example, the command **SELECT search(“a”)** selects all aromatic atoms, and the command **SELECT*on*search(“a”)** highlights them. Some commands, such as search(…), smiles(…), and find(…) can be applied to atom sets in Jmol math expressions. For example, **carbonyl** **=** **{1.1}.search(“C=O”)**, after which the variable *carbonyl* can be used in a SELECT command: **SELECT @carbonyl**. The find(…) command has broad utility, but in this context we will see it used for comparing any combination of 3D model and/or string data using SMILES or SMARTS. Thus, **x** **=** **{1.1}.find(“SMARTS”,“a”)** is synonymous with **x** **=** **{1.1}.search(“a”)**, and also we can have **{1.1}.find(“SMILES”,“C(C)OCC”)**, **“CCOCC”.find(“SMARTS”,“COC”)**, and **“CCOCC”.find(“SMILES”,“C(C)OCC”)**. The commands **SHOW SMILES** and **PRINT {molecule=1}.find(“SMILES”)** display SMILES strings—the first for the current selection; the second for the first molecule (in a model with more than one molecule).

### Jmol SMILES (Tables 1–3)

In terms of SMILES for small molecules, Jmol’s implementation is a superset of OpenSMILES (Table 1). Thus, all valid OpenSMILES strings are also valid Jmol SMILES strings. All of the basic aspects of OpenSMILES are part of Jmol SMILES, including:

- Allowed unbracketed element symbols include B, C, N, O, P, S, F, Cl, Br, and I. Jmol SMILES adds H to this list of allowed unbracketed atoms.
- Bracketed atom notation adheres to the required ordering *[*<*mass*>*symbol*<*stereo*><*hcount*><*charge*><*:class*>*]*, where <mass> is an optional atomic mass, *symbol* is an element symbol or “*” (unspecified atom, with unspecified mass), <*stereo*> is an optional stereochemical isomer descriptor given in Table 2, <*hcount*> is an optional implicit hydrogen atom count, <*charge*> is an optional formal charge in the form (−1, +1, −2, +2, etc.) or (–, +, – –, ++, etc.), and <:*class*> is an optional non-negative integer preceded by a colon.
- Possible aromatic elements, indicated in lower case, include b, c, n, o, p, s, as, and se. Depending upon the directive, however, any element other than hydrogen may be allowed to be aromatic. This set is specific to /open/ with or without /strict/.
- Connections (indicated as a single digit 0–9 or “%” followed by a two-digit number) with their optional bond type preceding them, must follow bracketed or unbracketed atom symbols immediately. Connections may span no-bond indicators (“.”). Jmol SMILES expands this to allow any positive number to be used as a connection number.
- Branches, indicated in parentheses, follow connections, with their optional bond type as the first character after the opening parenthesis.
- Bond types include -, =, # (triple), $ (quadruple), “:” (colon; aromatic, never significant), and “.” (period, indicating no connection), as well as the cis/trans double-bond stereochemical indicators/, and \. Single bonds between aromatic atoms indicate biaryl connections.

Jmol SMILES adds several more features as well, as shown in Tables 1, 2 and 3. These include more flexible formatting, processing “directives”, the atomic symbol Xx (used in quantum mechanics computational programs to indicate a reference point that is not part of the chemical structure), unlimited connection numbers, and more extensive handling of stereochemistry, including stereochemical designations for odd- and even-cumulenes, imines, and carbodiimides, as well as trigonal pyramidal, T-shaped, and see-saw molecular shapes. The bond notations ^nm- and ^^nm- indicate atropisomerism.

**Table 1:** Basic Jmol SMILES additions

Note	Notation	Meaning	Example	Explanation
a+	//*….*//	Comment	//* prod. by Jmol *//	Optional; application-dependent; no general function; removal does not affect processing
a+	<whitespace>	Allowed for formatting		Optional use of whitespace; removal does not affect processing of Jmol SMILES (however there may be aspects of Jmol SMARTS that require whitespace, these are application-dependent).
a+	/…../	Processing directive	/strict/c1cccccc1	Optional; if present, must precede molecule description; see discussion and Table 3
+	[Xx]	Dummy atom	[Xx]	The atomic symbol “Xx” represents a “dummy” atom that is present but not part of the actual chemical structure
+	<lower-case symbol>	Aromatic atoms	c1cocc1	Furan Any atom other than hydrogen may be indicated as aromatic. Note that only b, c, n, o, p, si, and se are allowed to be aromatic using the processing directives/open/or/strict/(see below).
+	%(n)	Unlimited connectivity	C%(102)CCC%(102)	Any non-negative number
+	a=a	Aromatic double bond	c1cc(O)=c(O)cc1	A specific double bond Kekulization, but still aromatic

In the first column, a indicates result may depend upon application; + indicates additions to OpenSMILES

**Table 2:**
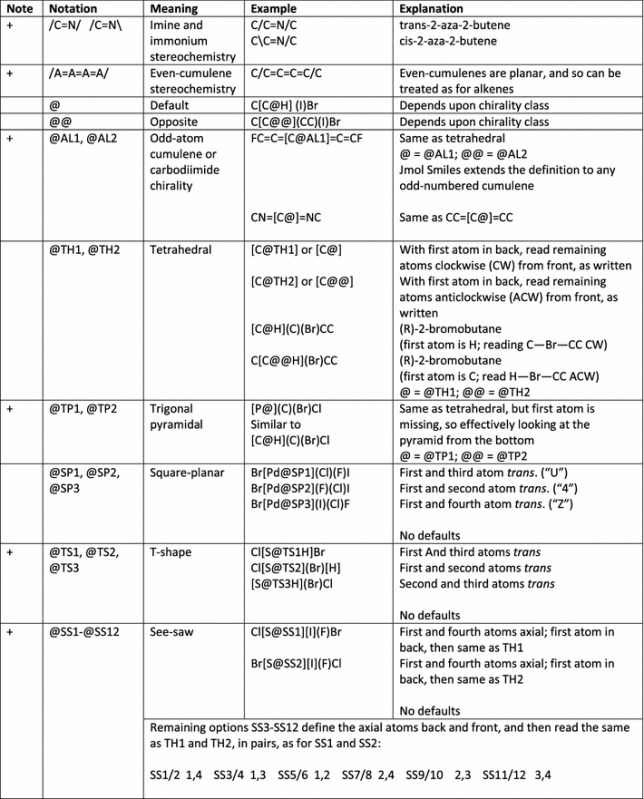

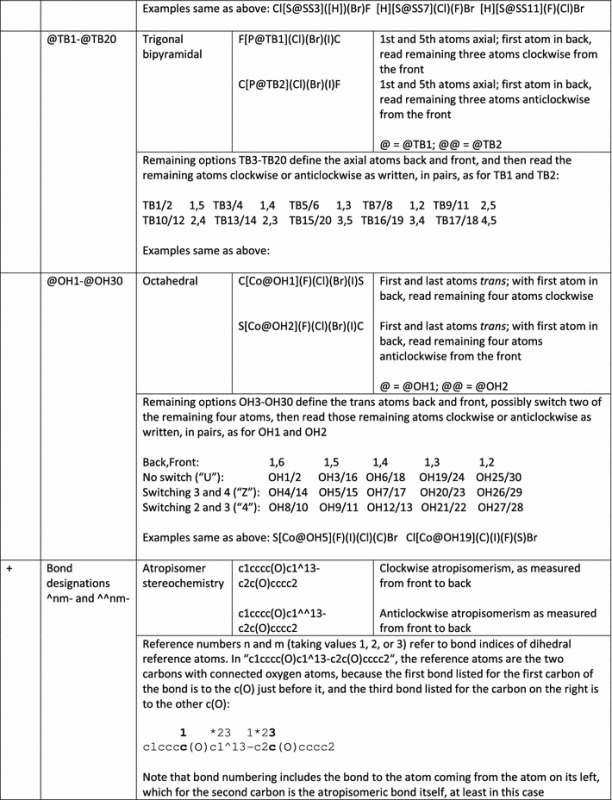
Stereochemical aspects of Jmol SMILES

In the first column, absence of a mark indicates same as OpenSMILES; + indicates additions to OpenSMILES

**Table 3:**
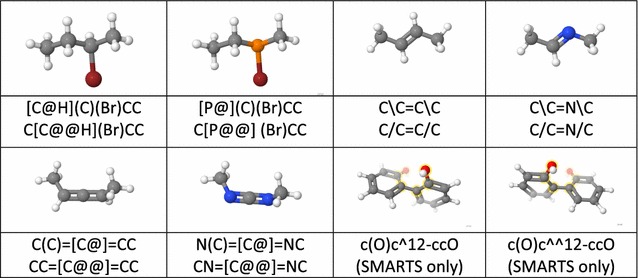
Examples correlating Jmol SMILES stereochemical notation with 3D structures, illustrating the similarity of new definitions to standard ones

Biaryl images are SMARTS matches; all others are SMILES descriptions. Equivalent alternative descriptions for the acyclic compounds. Note how switching of positions of atoms in the SMILES strings can result in switching of the stereochemical descriptor

### Jmol SMILES general additions (Table 1)

In terms of formatting, the only difference is that Jmol SMILES allows for comments and whitespace. Whitespace in Jmol SMILES simply allows more flexibility and a more human-readable string; comments allow annotation of the created strings with information about the program used to generate it or whatever is relevant to the designer of the system. In addition, Jmol SMILES includes an optional prefix, set off by matching forward slash characters, that gives directives to a processor that specify how the SMILES string is to be interpreted (see below). It is simple enough to convert these annotated Jmol SMILES strings to more standard SMILES. One simply strips out the directives, comments, and white space. Jmol itself simply strips out all comments in a preprocessing step and ignores all whitespace, as there is no context in Jmol SMILES where whitespace is relevant.

**Fig. 1:**
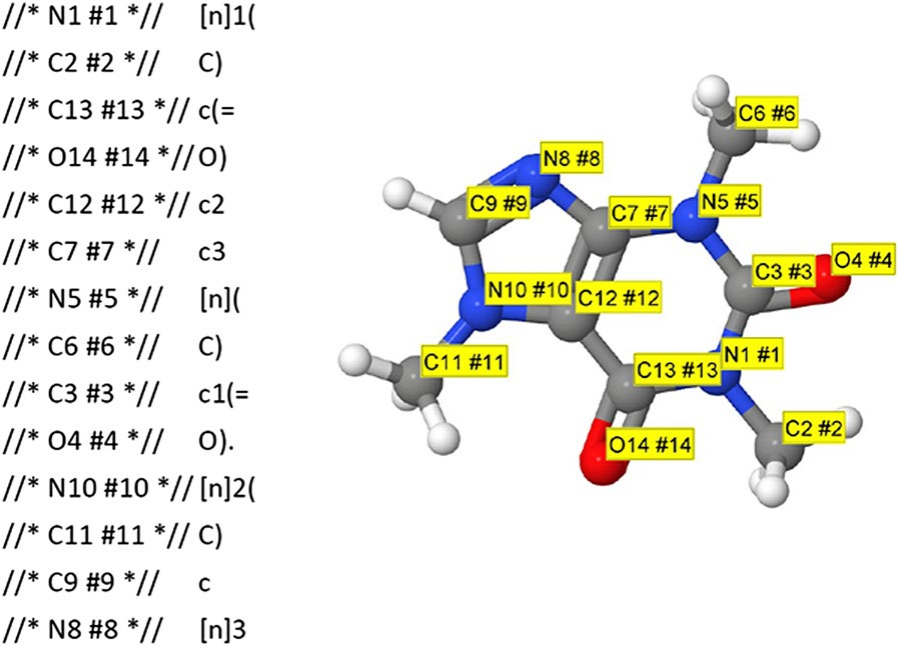
Using comments and white space to correlate a SMILES string with a 3D structure. This Jmol SMILES was generated using **LOAD $caffeine; SHOW SMILES/open,atomComments**

Comments in Jmol SMILES are set off as //* … *//. Their utility is illustrated with a simple example. The OpenSMILES representation of caffeine, from the Jmol commands **LOAD $caffeine; SHOW SMILES/open** is *[n]1(C)c(*=*O)c2c3[n](C)c1(*=*O).[n]2(C)c[n]3*. While useful, perhaps, what we are missing is a clear 1:1 correlation between atoms in our structure and atoms in the SMILES string. If instead, one issues in Jmol **SHOW SMILES/open/atomComments**, one gets the result in Fig. 1. The comments allow us to quickly correlate specific atoms in the structure with specific atoms in the SMILES string. We can see that the sequence N1–C2–C13–O14–C12–C7–N5–C6–C3–O4 is working its way clockwise around the six-membered ring, and N10–C11–C9–N8 are the added four atoms forming the five-membered ring, completing the structure.

The other additions shown in Table 1 simply broaden the range of applications of SMILES. Jmol SMILES allows for “dummy atoms” such as those sometimes found in quantum mechanics calculations to be introduced as [Xx]. They have atom number 0 and match only [Xx] and [#0], not “any atom.” The %(n) syntax allows connection numbers greater than 99. While having 100 open connections may seem impossible, and using large numbers is certainly not recommended in general, this feature is included at this time because it is of use in extensions of Jmol SMILES to be described in a future publication. Jmol SMILES allows for the option of more atoms being aromatic, for example when an aromaticity model does not involve bonding analysis or electron counting.

Finally, by allowing for double bonds between aromatic atoms, we can specify that double bonds in the pattern must also be present in the model or SMILES string being compared. That is, a successful match requires a specified Kekulé form of an aromatic system. It can be used to check to see if models from two different sources have the same Kekulé form. For example, 2-methylpyridine models retrieved from NCI/CADD and PubChem have different Kekulé forms. We need aromaticity models to compare them, but we still might want to distinguish them. The Jmol SMILES string [n]1ccccc1(C) will match both, but [n]1=cc=cc=c1(C) will match only the one from PubChem.

### Jmol SMILES stereochemistry (Tables 2, 3)

Jmol SMILES fully implements all OpenSMILES stereochemistry designations, including the restriction that double-bond designations / and \ must be matched. In addition, Jmol SMILES straightforwardly expands cis/trans double bond stereochemistry to cover even-numbered atom cumulenes, imines, and immonium ions. Similarly, Jmol SMILES extends standard allenic stereochemistry to odd-numbered cumulenes and carbodiimides. Jmol SMILES supplements tetrahedral (TH), square planar (SP), trigonal bipyramidal (TB), and octahedral (OH) stereochemistry notations with notations for trigonal pyramidal (TP, covering chiral phosphines and sulfoxides, for example), T-shaped stereochemistry (TS), and seesaw (SS). Finally, Jmol SMILES adds the single-bond stereochemistry designations ^nm- and ^^nm- to indicate atropisomerism. Examples of Jmol SMILES notation for imines, carbodiimides, phosphines, and biaryls are given in Table 3.

A reader with knowledge of organic chemistry R/S stereochemical nomenclature will find a familiar pattern in these explanations, namely that @ generally involves putting an atom in the back and reading the remaining atoms clockwise, in sequential order of left to right. Thus, if the first atom is the lowest priority atom (often H), and the remaining atoms are listed from highest to lowest—for example, [C@H](Br)(CC)(C)—then @ is “R” (H in back; read left-to-right highest to lowest), while @@ is “S”. Readers more familiar with standard SMILES explanations of this notation or like the idea that the “at” symbol has an inherent anticlockwise sense to it, may wish to replace “front” with “back” and “clockwise” with “anticlockwise” with no change in meaning.

### Jmol SMILES directives (Tables 4, 5)

Jmol SMILES input and output can be configured for several different nuanced dialects of SMILES. This is done by prefixing a search with directives marked off with slash marks (Table 4). These directives are not case-sensitive. Thus, **/noaromatic/** and **/NoAromatic/** both mean the same thing. Multiple directives may be placed between slash marks. No separation is required, but some sort of separator is recommended—for example, **/noAromatic,noStereo/**. Applications may add their own application-specific directives.

**Table 4:** Jmol SMILES directives

Prefix	Meaning	Jmol example
/open/	Use the OpenSMILES aromaticity model; required for Jmol SMILES matching of atom class	LOAD $quinone SELECT smiles(“/open/c1ccccc1”) (will return “0 atoms selected” because OpenSMILES does not consider quinone to be aromatic)
/strict/	Use electron counting to define aromatic ring using the Hückel rule (Jmol application default)	LOAD :cyclobutadiene SELECT smiles(“/strict/c1ccc1”) will return “0 atoms selected”
/noAromatic/	Ignore upper/lower case indicators of aromaticity; indicated double bonds must match exactly	/noAromatic/C1CCCCC1
/noStereo/	Ignore differences in stereochemistry at chirality centers and double bonds	/nostereo/C{[C@H]}(O)CC=C
/invertStereo/	Reverse stereochemistry match result (chirality centers only, not double bonds)	/invertstereo/C{[C@@H]}(O)CC=C
/noAtomClass/	Disregard atom class designations when matching	/noAtomClass/C[C:1]CC

**Table 5:** “Open” versus “strict” interpretation of aromaticity

Aromatic?						
**Open**	Yes	No	No	Yes	Yes	Yes
**Strict**	No	No	No	Yes	Yes	No

The Jmol SMILES directives **/open/** and **/strict/** relate primarily to the aromaticity model assumed in the SMILES string that is to be processed by the application’s SMILES matcher. This is important, because different SMILES generators and parsers have different aromaticity models. These directives allow appropriate interpretation of SMILES using their original models. Examples of differences in these models are shown in Table 5. The first of these, **/open/**, uses the OpenSMILES definition of aromaticity, which involves a version of the Hückel 4n + 2 rule that allows for inclusion of ring atoms doubly bonded to acyclic atoms, provided those atoms are not more electronegative than carbon. The **/strict/** directive, which is the default model for Jmol 14.6, goes one step further, applying a stricter (organic chemist’s) definition of aromaticity, both requiring three-dimensional planarity^1^ and also not allowing double bonds to exocyclic atoms. Within this model, 3,6-dimethylidenecyclohexa-1,4-diene and quinone are nonaromatic because they are not cyclic pi systems, cyclobutadiene is nonaromatic because is not 4n + 2, and 1-oxothiophene is nonaromatic because it is nonplanar. Note that /strict/ and /open,Strict/ are equivalent.

The directive **/noAromatic/** indicates that no aromaticity checks of any kind should be made. Thus, C1CCCCCC1 and c1ccccc1 both would match both benzene and cyclohexane. The bond type “:” would be considered simply to be “unspecified.” This directive is useful when it is not desired to make any aromaticity assumptions at all or to specifically test for one Kekulé version and not do any aromaticity tests.

Directives **/noStereo/** and **/invertStereo/** are very useful because they allow re-use of SMILES strings for different types of stereochemical matches without having to remove or switch the stereochemical designations in the strings themselves, which can be quite complicated. The directive **/noStereo/** simply ignores all stereochemistry indicated in the SMILES string, including both stereochemistry at chirality centers as well as cis/trans double-bond stereochemistry. The directive **/invertStereo/** inverts all chirality designations, allowing efficient checking for enantiomers. Finally, the directive **/noAtomClass/** instructs the parser to disregard atom classes when creating the molecular graph for matching.

### Jmol SMARTS (Tables 6, 7)

The Jmol SMARTS dialect expands significantly on the OpenSMARTS language. Given below is a full description of Jmol SMARTS, not simply a list of additions to that language. All differences to OpenSMARTS are indicated. A discussion of compatibility issues with OpenSMARTS and Daylight SMARTS is given later in this paper.

**Table 6:** Jmol SMARTS atom selection primitives

Note	Notation	Meaning	Example	Explanation
S-	*	Any atom	*1**1	Any three-membered ring
S-	a	Any aromatic atom	a1aaaa1	A five-membered aromatic ring
S-	A	Any non-aromatic atom	AAAA	A chain of at least four nonaromatic atoms
+-	H	Hydrogen	OH	All OH groups; SMARTS only
a+	#-<n>	Negative of atom number	[#-36]	Atom number 36, as defined by the application. (In Jmol, this corresponds to “@36” or “atomno=36”.)
+	<n>?	Mass number or unspecified mass number	[12?#6]	Carbon that isn’t explicitly C13 or C14
			[0?]	Atom with unspecified mass
			[!0?]	Any atom with mass specified
S	X<n>	Total number of connections	[X2]	This includes all implicit hydrogens, whether in a molecule or SMILES string
+	d<n>	Number of non-hydrogen connections	[n;d3]	Aromatic trivalent nitrogen with no attached H atom. Note that [nd3] would be read as an aromatic neodymium with an atomic mass improperly positioned after it
S	D<n>	Number of explicit connections	[#6D3]	Carbon atoms with exactly three connections (either in a molecule with bonds to three atoms or in a SMILES string with three explicit atoms connected to it
S	h<n>	Number of implicit hydrogens	[C;h2]	A methylene group written as “C” or [CH2] in a SMILES string; a methylene carbon atom from a PDB file or other file that does not contain hydrogen atoms. (In Jmol, for example, a non-NMR PDB file loaded before issuing **SET pdbAddHydrogens TRUE**.)
S	H<n>	Total hydrogen count (sum of attached [H] and implicit)	[CH3]	A methyl group
			[H0]	No attached hydrogens
			[!H0]	At least one attached hydrogen
a*	x<n>	Total number of bonds that terminate on ring atoms	[x1]	A non-ring atom that is connected to a ring
			[x0]	No ring connections
			[!x0] or [x]	At least one ring connection
a*	R<n>	Ring membership	[R]	A ring atom
			[!R]	A non-ring atom
			[R2]	An atom in exactly two rings
a*	r<n>	Ring size	[O;r3]	An oxygen in a three-membered ring
			[O;!r3]	An oxygen that is not in a three-membered ring
*+	r500	Five-membered aromatic ring	[n;r500]	Aromatic nitrogen in a 5-membered aromatic ring (not an aromatic nitrogen in a 500-membered ring)
*+	r600	Six-membered aromatic ring	[n;r600]	Aromatic nitrogen a 6-membered aromatic ring (not an aromatic nitrogen in a 600-membered ring)
S	v<n>	Total bond order (valence)	[C;v3]	Total bond count (note that ill-defined resonance structures such as proteins without hydrogen atoms will overestimate valence for arginine sidechains
+	Xxx#nn^c.yyyy#mm	PDB residue name#number^insertionCode. atom name#atomic number		For PDB data:
			[ALA.C]	Carbonyl carbon of all alanines
			[ILE#35.*]	All atoms in ILE35
			[#35.*]	Residue 35
			[*.CA&!CA.CA]	Alpha carbons (not calcium ions)
			[*.CA#6]	Just alpha carbons (atomic number specified) Note that all matches are by name only, not by analyzing substructure; atom name may include “.” or “*”; residue name may contain “*”
+	=<n>	Atom index	[=22]	Atom with atom index 22, however that is defined by the application
+	“xxx”	Atom type	[“7”]	However that is defined by the application; for example, in Jmol, [“7”] is a carbonyl carbon atom after assignment by MMFF94
	$(…)	Nesting	[$(aaN)$(aaaC)]	An aromatic atom that is both ortho to an amino group and meta to a methyl group
+	$(select ….)	Processor-specific selection phrase	[$(select atomno=x)]	Selects the atom with atom number equal to the value of the Jmol variable x
+	$<n>(pattern)	A specific number of occurrences of pattern	C[$3(C=C)]C	Nonterminal conjugated triene
+	$min-max(pattern)	A variable number of occurrences of pattern	C[$2-3(C=C)]C	Nonterminal conjugated diene or triene
+	$<varName>	A predefined variable	[$a1]	Replaces “[$a1]” with whatever $a1 is defined to be (see below)

Note that all patterns in Tables 1 and 2 are also part of Jmol SMARTS

S indicates same as OpenSMARTS; + indicates additions to OpenSMARTS; * indicates modified definition for Jmol SMARTS; “a” indicates result may depend upon application; - indicates does not need brackets

**Table 7:** Jmol SMARTS non-primitives

Notes	Notation	Meaning	Example	Explanation
+	{…}	Selection set; include only these atoms in the final atom selection	{[#6]}C=O	Select all alpha carbons
+	A(.d:x-y)B	Distance from connected atoms A and B within range x–y	C(.d:1.5-1.6)C	All aliphatic carbon–carbon bonds that are between 1.5 and 1.6 Å long
+	A(.d:!x-y)B	Optional “!” indicates “not in this range”	C(.d:!1.5-1.6)C	Select all CC bonds not in the range 1.5-1.6
+	A(.a:x-y)BC	Angle A–B–C within range x–y	C(.a:115-125)CC	All CCC with angle between 115 and 125 degrees
+	A(.t:x-y)BCD	Dihedral A–B–C–D within range x–y, where x and y are in the range −180 to 180	{[CH3]}(.t:50,70)CC{[CH3]} ||{[CH3]}(.t:-50,-70)CC{[CH3]}	Find gauche methyl groups and select just them
+	$R1=“xxx”;	Replacement variables	.$R1=“C=O”; $(*[$R1])	Same as $(*C=O)
D+	(SMARTS.SMARTS) (SMARTS).(SMARTS)	component-level grouping (with definition of a component determined by/groupByModel/and/groupByMolecule/directives in Jmol)	(F.O)	Unconnected F and O in the same component
			(F).(O)	F and O in different components
			(*).(*)	Only select atoms if there are multiple components

D indicates same as Daylight SMARTS; + indicates additions to OpenSMARTS

### Jmol SMARTS atom primitives (Table 6)

Jmol SMARTS is closely related to OpenSMARTS, involving 13 additional atom primitives and two modified primitives (Table 6). This table comprises the full set of atom primitives in Jmol SMARTS. Several of these added primitives in Jmol SMARTS were critical in the development of an MMFF94-based minimization that uses SMARTS for atom typing. As in OpenSMARTS, selected upper- or lower-case element symbols as well as *, **a**, and **A** do not need square brackets. Jmol SMARTS adds **H** to this list. Without brackets, CH is simply the same as C[H] and means “a carbon and its attached H,” whereas [CH] means “a carbon with exactly one attached H” (that is, the C only, not the H atom).

Thus, in OpenSMARTS, [D2] matches any atom with two explicit connections. This does not distinguish between hydrogen and non-hydrogen atoms. Jmol SMARTS adds **[d2]** to mean “exactly two non-hydrogen connections,” and in Jmol the command **SELECT search(“[C;d2]”)** selects for aliphatic carbons in the loaded atoms with exactly two *non*-*hydrogen* connected atoms. It should be noted that these atoms will be found regardless of whether the model actually has hydrogen atoms or not. This is an important distinction, because some models used in Jmol have hydrogen atoms (those from NCI/CADD), and some do not (some of those from RCSB). The new primitive **[<n>?]** selects for atoms with either an atomic mass of n or no indicated atomic mass. Like atom mass itself, this primitive must immediately precede an atom symbol. So, for example, [12?C] matches aromatic ^12^C or C with no indicated isotope (a common situation), but not ^13^C or ^14^C.

The ring selectors **[r500]** and **[r600]** are particularly useful, as they specify a 5- or 6-membered *aromatic* ring atom, respectively, which is not something that is supported in OpenSMARTS. (Note that in OpenSMARTS, [c&r5] could be an aromatic carbon in a benzene ring, as long as there is a fused 5-membered ring (as in indene) not specifically a carbon atom in an aromatic 5-membered ring.) This coopting of [r<n>] for large n technically is not compatible with OpenSMARTS, but since it is basically inconceivable that an actual ring of size 500 or 600 would ever be searched for using Jmol SMARTS, it is felt that this is not a practical problem.

Finally, Jmol SMARTS patterns also allow for referencing PDB “residue.atom” notation: **[ala.C]**, [ala.*], and [*.C]. This feature is strictly a lexical match, not a substructure search, and does not allow searching for the residue or atom name “*” itself or for residue names containing a period character. No such residue or atom names exist in the PDB. The residue component may include up to three parts, including residue name, number, and insertion code as “resName#resNum^insCode”. The atom component may include PDB atom name and atomic number as “atomName#atomicNum”. The atomic number can be used to distinguish calcium, **[.CA#20]**, from alpha-carbon, **[.CA#12]**. An example of a fully elaborated PDB primitive would be **[G#129^A.P#15].** Any of the five references resName, resNum, insCode, atomName, or atomicNum, may be omitted or indicated as the wild card “*”. Thus, the critical distinguishing characteristic of Jmol SMARTS PDB notation is only the period itself.

Three additional atom primitives allow for atom selection that is application specific. So, for example, **[=0]** selects for the atom the application assigns index 0 to. In Jmol, [=0] would refer to the first atom in the Jmol atom array, ({0}). The notation **[“x”]**, with quotation marks, selects for atom type “x”, however that has been defined in the application. In Jmol, atom types will default to the atom’s name, such as “H12”, but can be set by a specific file reader or by the user or by an MMFF94 minimization or partial charge calculation.

Jmol SMARTS allows for nested (aka “recursive”) searches. This option allows embedding a full SMARTS string as an atom primitive, selecting the first atom only. So, for example, [$(cc[OH])] is “the aromatic carbon atom ortho to an aromatic OH, and in Jmol **SELECT on search(“[$(HccOH)]”)** highlights the two ortho hydrogens of a phenol.

The general pattern **[$(select …)]** allows for a hook into application-specific selection methods. For example, in Jmol **SELECT atomno<10** selects all atoms with atom number less than 10. **SELECT search(“…”)** selects using a SMARTS pattern, and so **SELECT search(“[$(select atomno<10)]Br”)** does the same, but limits the result to atoms connected to bromine. The [$(select…)] notation thus allows both a potentially huge expansion of SMARTS capability as well as potentially bringing into an application’s native search language all the rich capability of SMARTS, if they are not already present. Notice that, if implemented in an application, this option may require that whitespace not be unilaterally stripped from a Jmol SMARTS pattern prior to processing.

The last three of the entries in Table 5 allow for a variable number of patterns and for substitution of predefined variables. In Jmol, these variable substitutions are carried out as preprocessing steps, in a purely lexical fashion. They do not in any way improve processing time. (See Additional files 1, 2 for examples.)

The Jmol SMARTS dialect includes all bond primitives of OpenSMILES as well as ~ (any bond) and @ (any aromatic bond). It does not implement the “direction or unspecified” primitives of OpenSMARTS (/? and \?) for two reasons. First, when working with a 3D model, all double bonds are specifically *E* or *Z*. Additionally, Jmol SMILES is based on OpenSMILES and thus already requires that / and \ be matched properly. So FC=C/Cl is not a valid Jmol SMILES string, and a search in it for F/?C=C/Cl therefore would not be relevant.

Jmol SMARTS implements all logical operations of OpenSMARTS, both in atom primitives and bonds. These include the standard operations “!” (NOT), “&” (AND), and “,” (OR) as well as the special “low precedence” AND operator “;”. The low precedence AND operator makes up for the fact that SMARTS does not implement parentheses in logical operations, allowing, for example, for [S,O;X2] to be parsed as “(aliphatic sulfur or oxygen) with two connections”, in contrast to [S,O&X2], which would mean “sulfur or (oxygen and two connections)”. Perhaps WITH would be a better description than AND for this low-precedence version of AND. The default operation between two primitives is &. Thus, [S,OX2] is the same as [S,O&X2], not [S,O;X2].

Jmol SMARTS allows for a larger-scope “or” logic using “||”. This notation is strictly a lexical convention carried out in a pre-processing stage. For example, **C=[O,S] || N=[O,S]** indicates to run two separate SMARTS matches and then OR their results. In Jmol this amounts to selecting all atoms resulting from either search.

### Additional Jmol SMARTS features (Table 7)

Several non-primitive Jmol SMARTS options extend OpenSMARTS. They are presented in Table 7.

In general, SMARTS matching is used in a binary sense, returning TRUE if there is a match, or FALSE if not. In addition, in some contexts, it is valuable to know which subset of atoms in a model are the atoms that match. But there is another valuable possibility. Once a match is found, it could be especially valuable if some *subset of those matched atoms* is identified. This adds significant power to a SMARTS search, as it can answer questions such as “What atom is next to atom X in this pattern?” This more nuanced capability in Jmol SMARTS is provided using curly braces, for example, **{C}C=O**. The overall pattern is first matched, then only those atoms that are within braces are actually identified. Thus, CC=O matches all atoms of an aliphatic carbonyl group and its associated alpha carbons, but {C}C=O returns only the alpha carbons of carbonyl groups, and {C}[CH]=O returns only the alpha carbons of aldehydes. This allows very specific atom selection based on the identity of groupings of atoms. Any number of selection braces can be present in a Jmol SMARTS pattern. Thus, **select on search(“{c}1c{c}c{c}c1[OH]”)** in Jmol selects for the ortho- and para-carbons of phenol.

Conformational matching, involving ranges of distance, angle, and torsion measurements (including improper torsions), have also been of interest to Jmol users. Such matching is possible using Jmol SMARTS. This is done using the notation **(.d:)**, **(.a:)**, and **(.t:)**, respectively. A range of values is included after the measurement type. **C(.d:1.30-1.40)C**, for instance, matches aliphatic carbon–carbon bonds in the range of 1.30–1.40  Å. Valid separators include comma and hyphen. Thus, **C(.d:1.30-1.40)C** and **C(.d:1.30,1.40)C** are equivalent. Bond angles range from 0 to 180, as might be expected; torsions range from −180 to 180. Positioning of these notations should be after the atom they refer to, so that the order of attributes to a SMARTS atom is either atom–connections–branches–measurements–bond or atom–connections–measurements–branches–bond. For example: **C(.d:1.30-1.40)=C,** not C=(.d:1.30-1.40)C; **C1(.d:1.30-1.40)C** not C(.d:1.30-1.40)1C. Any number of “OR”ed ranges can be given, separated for clarity preferably by commas. For example: {*}(.t:-170,-180,170,180)C=C{*} selects for vinylic atoms that are trans-related. In addition, “not this range” can be indicated using **“!”**, so that an equivalent description to the above would be {*}(.t:!-170,170)C=C{*}. Ranges should be selected to have some width appropriate to an application.

The default in terms of specifying which atoms are involved in measurements is simply “the next N atoms in the string,” where N is 1, 2, or 3, respectively. This sequencing is strictly lexical and is entirely irrespective of chains. So, for example, the highlighted atoms are measured in the order shown, from left to right, in each of the following measurements: **C**(.a:0,120)**C**(**C**)C, C**C**(.a:0,120)(**C**)**C**, and C**C**2(.a:0,120)(**C**).**C**2.

For more complicated patterns, one can designate the specific atoms in the measurement using a numeric identifier after the measurement type and then repeat that designation immediately after each of the target atoms. For example, the following will target a range of unusually low bond angles across the carbonyl group in the three-atom backbone of a peptide, CA–C–N: [*.CA](.a1:105-110)C(.a1)(O)N(.a1). In this way, there is no requirement that measured atoms be connected. Distances can be through-space; angles need not be bond angles; torsions can be improper angles. These numbers may be re-used, as for connection numbers.

Jmol SMARTS allows the use of any number of predefined variables. These are separated by semicolons and indicated prior to the actual SMARTS pattern (but after any directives). Variables may refer to other variables, as long as the variables referred to are defined previously. So, for example, the following construction is allowed: **$R1=“[CH3,NH2]”;$R2=“[OH]”; [$([$R1]),$([$R2])]**, meaning “a CH3, NH2, or OH group.” Variable replacement is best carried out by a Jmol SMILES parser immediately following removal of comments but prior to any other parsing.

Jmol SMARTS implements the Daylight SMARTS “component-level grouping” functionality of the form (…).(…). Though of rather specialized use, it would, for example, allow highlighting the diene in one molecule and the dienophile in another with ({C=CC=C}).({C=C}C=[!C]). The following Jmol SMARTS pattern will match any OH group in a component that has at least two carbonyl groups: (C=O.C=O).{OH}.

### Jmol SMARTS directives

Just like Jmol SMILES, Jmol SMARTS matching can be tuned to specific modes of searching in terms of different standards. This is done using the same directives described above for Jmol SMILES. For example, in Jmol, the commands **LOAD :cyclobutadiene; SELECT search(“/strict/c”)** loads a 3D structure of cyclobutadiene from PubChem and reports “no atoms selected”, because cyclobutadiene is strictly not aromatic.

### Jmol SMARTS compatibility issues

Jmol SMARTS does not include the OpenSMARTS unspecified designations /? or \?. In addition, Jmol SMARTS does not implement the unspecified stereochemistry notation @..?, as these have not proven relevant to 3D molecule searching. Jmol SMARTS implements “.” as absolutely “not connected” rather than “might not be connected.” Jmol SMARTS is not an extension of Daylight “reaction SMARTS” [4], although it does allow for matching atom classes, which are generally only relevant in a reaction context, and Jmol as an application can read reaction SMILES, but simply reads “>>” as the not-connected symbol “.”.

Jmol SMARTS implements ring-membership primitives [r<n>] and [R<n>] within the OpenSMARTS framework using a simple ring membership model as “within any ring of size n” and “the number of rings containing the atom”, respectively. This involves no concept of *smallest set of smallest rings* (SSSR). An application implementing Jmol SMARTS is free to limit ring size in ring membership determinations. In Jmol, for performance sake, the maximum ring size that will be checked by default is 8, but that is increased simply by having any check for any ring larger than 8. For example, for indole, which contains a five-membered ring fused to a six-membered ring, so three rings total, of size 5, 6, and 9, **select on search(‘[R2]’)** will select the two atoms in the fusion, because the 9-membered ring is not checked. However, while **select on search(‘[R2&r9]’)** will select all the atoms not involved in the ring fusion, since now three rings will be found, and those central two atoms will be considered to be in three rings, not two.

### Jmol application-specific directives (Table 8)

Table 8 lists application-specific directives for Jmol 14.6. Upon SMILES generation, **/atomComments/** adds comments indicating which atoms in the structure map to which atoms in the SMILES string, and the **/hydrogens/** directive indicates that all hydrogen atoms are to be given explicitly. The **/topology/** directive creates a SMILES string that shows * for all atoms and indicates no bond types. It can be used for matching ring and chain patterns without regard to specific atoms or bonds.

**Table 8:** Jmol application-specific directives

Note	Directive	Meaning	Jmol example
G	/atomComments/	Add comments when generating SMILES strings that indicate the correlation between SMILES atoms and Jmol atoms	PRINT {*}.find(“SMILES/atomComments/”) SHOW SMILES/atomComments
G	/hydrogens/	Makes all hydrogens explicit in generating a SMILES string	LOAD $benzene PRINT {*}.find(“SMILES/hydrogens/”) *c1([H])c([H])c([H])c([H])c([H])c1[H]*
G	/topology/	Generate SMILES strings that represent all atoms as “*”, thus allowing SMARTS pattern matches without regard to specific elements	LOAD $indane SHOW SMILES/topology *1*2*****2**1
P	/firstMatchOnly/	Return only the first match, not all occurrences of a match	LOAD $heptane SELECT ON search(“/firstMatchOnly/C”) *1 atom selected*
P	/groupByMolecule/	3D components are grouped by covalently-bonded sets (Jmol default)	LOAD “$carbetapentane citrate” SELECT on search(“/groupByMolecule/(C=O.C=O)”) (highlights the carbonyl groups of the citrate ion only, because carbetapentane has only one carbonyl group, and the indicated grouping requires that both be in the same component)
P	/groupByModel/	Consider each model in Jmol to be one component, regardless of how many covalently disjoint sets it contains	x = search(“/groupByModel, firstMatchOnly/(C).(C)”) (returns an atom set containing the first nonaromatic carbon of each model when there are two, or no atoms if there is only one model)
GSP	/aromaticDouble/	Double bonds between aromatic atoms must match explicitly for 3D molecule comparison—SMILES strings and SMARTS patterns with = between aromatic atoms will set this flag automatically during processing	LOAD files “$1,2-dihydroxybenzene” “:1,2-dihyroxybenzene” PRINT {1.1}.find(“SMILES”,{2.1}) == {1.1} *true* (because both NCI and PubChem have structures for these two compounds) PRINT {1.1}.find(“SMILES/aromaticDouble/”,{2.1}) == {1.1} *false* (because the two structures have different double-bonding patterns)
GSP	/aromaticPlanar/	Carry out a 3D analysis to define aromatic atoms	LOAD $quinone SELECT smiles(“/aromaticPlanar/c1ccccc1”) (will return “6 atoms selected” because Jmol’s default is to consider the planar ring of quinone to be aromatic)
GSP	/aromaticDefined/	Bonds to be matched as aromatic are already marked as such; make no attempt to determine aromaticity in the structure	LOAD :benzene CONNECT {*} {*} aromatic modify CALCULATE aromatic SELECT smiles(“/aromaticDefined/c1ccccc1”) (aromatic atoms have been pre-calculated)

G for Jmol SMILES generation; S for Jmol SMILES (full molecule) matching; P for Jmol SMARTS pattern matching

Three directives are specific to SMARTS matching. The **/firstMatchOnly/** directive tells the Jmol SMARTS processor to stop after one successful match. The Jmol application-specific directives **/groupByModel/** and **/groupByMolecule/** (the Jmol default), govern how component-level grouping is done.

Aromatic models are important for SMILES generation and matching. The directive **/aromaticPlanar/**, which was the Jmol default through Jmol 14.5, is also available. This directive avoids any Hückel analysis and is based instead solely on three-dimensional ring planarity (see footnote 1), without respect to electron counting. The /**aromaticPlanar**/ option allows planar sp2-hybridized systems such as quinone and cyclobutadiene to be considered aromatic and allows the finding of aromatic rings in structures that may or may not indicate any multiple bonds, such as the results of many quantum mechanics calculations and structures saved in XYZ and PDB formats. In addition, the directive **/aromaticDefined/** indicates that all aromatic atoms in the model to be investigated are explicitly set already, and thus no aromaticity model is necessary. This directive could be used in Jmol when a structure is loaded from a file that includes explicit bond aromaticity, such as SDF query files, where bond type 6 is “aromatic single” and bond type 7 is “aromatic double” [19]. Both **/strict/** and **/aromaticDouble/** are used in Jmol’s MMFF94 [20, 21] determination of atom types.

## Results and discussion

### MMFF94 atom typing

One of the first applications of Jmol SMARTS was in Jmol’s implementation of the molecular mechanics minimization package MMFF94. For this method, each atom must be assigned a specific atom type, with identifications such as “general 5-ring C (imidazole)” and “alpha aromatic 5-ring C”. The MMFF94 program itself uses an elaborate sequence of logical steps to discover each of 82 distinct atom types for each atom in a structure, one at a time. Rather than attempting to implement this complicated algorithm in Java de novo, it was decided to have Jmol instead use SMARTS to do this task, scanning through types rather than atoms and identifying all atoms of a given type at once (and automatically skipping checking for types for elements that are not in the structure. The key is to go through a list of SMARTS checks in a very specific order. A full list of SMARTS tests used by Jmol for MMFF94 atom typing is given at SourceForge [21].

Table 9 shows the sequence of Jmol SMILES checks specifically for sulfur. All sulfur atoms are assigned one of atom types 15, 16, 17, 18, 44, 72, 73, or 74. The order of these tests is important. For example, the test for sulfur doubly bonded to carbon (atom type 16; $([SD1]=[#6D3]), must be done before the test for terminal sulfur (atom type 72; $([SD1][#6]). This works because once the algorithm finds an atom type match, no further tests are needed, and it exits. The process is made more efficient by grouping tests by element and valence and only running tests on element/valence combinations that are present in the compound. In addition, a single test can test for several environments at the same time. For example, the SMARTS search $([SD3]([OD1,ND2])([#6,#7D3,#8D2])[#6,#7D3,#8D2]) tests for sulfoxides, sulfonimides, and all related substitutions of carbon by N or O, all of which are categorized as atom type 71. Notice the efficient use of [r500] for thiophene sulfur.

**Table 9:** MMFF94 atom types for sulfur expressed in Jmol SMILES notation

18 SULFONAMIDE S	[$([SD4]([OD1,ND2])[OD1,ND2]),$([SD3](=C)([OD1,ND2])[OD1,ND2])]
17 SULFOXIDE S (also S(=O)[N])	[$([SD3]([OD1,ND2])([#6,#7D3,#8D2])[#6,#7D3,#8D2])]
73 SULFUR IN SULFINATE	[$([SD3]([OD1,SD1])[OD1])]
44 S IN THIOPHENE	[sD2r500]
15 THIOL, SULFIDE	[$([SD2](-*)-*)]
74 SULFINYL SULFUR, C=S=O	[$([SD2]([CD3])[OD1])]
72 THIOCARBOXYLATE S	[$([SD1][CD3][SD1])]
16 S DOUBLY BONDED TO C	[$([SD1]=[#6D3])]
72 TERMINAL SULFUR ON SPO, SCO, SSO	[$([SD1][#15,#6,#16][OD1])]
72 TERMINAL SULFUR ON C	[$([SD1][#6])]
72 TERMINAL SULFUR ON P or S	[$([SD1][#15,#16])]

### Practical examples

 Going back to the questions posed in the introduction to this paper, I now provide eight practical examples of Jmol SMILES matching and Jmol SMARTS searching within Jmol that are derived largely from Jmol user community requests for functionality.

#### 1. *Do these two structures and/or SMILES strings match?*

SMILES strings are often used for database look-up using simple string-based algorithms. In order for that to work, the SMILES string of interest must be expressed identically to one stored in the database. Basically, this means that it must be produced by the same algorithm used to produce the database’s own SMILES keys. The process of converting a generic SMILES string to a unique form is called “canonicalization.” Since SMILES generator programs at different databases differ, the resultant canonical SMILES strings from different databases can be different as well. For example, for acetaminophen, database look-ups from PubChem and NCI/CADD, as well as drawing the structure using JSME [22] give the distinctly different canonical SMILES shown in Table 10.

**Table 10:** Different canonical SMILES representations of acetaminophen

Service	“Canonical” SMILES	
PubChem	CC(=O)NC1=CC=C(C=C1)O	
NCI/CADD	C1=C(NC(=O)C)C=CC(=C1)O	
JSME	CC(=O)Nc1ccc(O)cc1	

Canonicalization can be useful; it allows a program to match structures using simple string matching. Interestingly, in the context of 3D structure matching in Jmol, given a single target 3D structure and a SMILES string, a pair of 3D structures, or a SMILES string and a 3D structure, there is no particular need for canonicalization. We simply compare two 3D sets of atoms and bonds, without ever generating two SMILES strings for comparison. (If a canonical SMILES is desired in Jmol, the command **SHOW chemical SMILES** can be used to pass the request to NCI/CADD for remote processing.)

Jmol’s **find()** function allows simple comparison of SMILES strings and/or 3D structures, regardless of their source. The syntax is as simple as ***A.find(“SMILES”, B)*** where ***A*** and ***B*** can be two SMILES strings or two models, such as {2.1}, representing “all the atoms in the first model in the second file,” or ({0:10}), (parentheses significant) meaning the first 11 atoms in the collection, regardless of model. For example, the Jmol command **LOAD files “$caffeine” “:caffeine”** loads the caffeine molecule from both NCI/CADD and PubChem. After that command, there are 48 atoms in an array behind the scenes in Jmol. The first 24, referenced as ({0:23}) or as {1.1}, are from NCI/CADD ($caffeine); the next 24, referenced as ({24:47}) or {2.1}, are from PubChem (:caffeine). Following this, **PRINT {1.1}.find(“SMILES”,{2.1})**, meaning “print the result of finding the second model “in” the first model using SMILES, reports *({0:23})*, indicating that all 24 atoms of the NCI/CADD structure were found. Alternatively, if we run a different function, we can find the 1:1 atom correlation between the two models: **PRINT compare({1.1},{2.1},“map”).format(“JSON”)**, which reports: [ [0,37],[8,36],[6,35],[7,30],[2,28],[11,29],[9,26],[1,27],[4,24],[13,25],[3,33],[12,34],[10,32],[5,31] ]. If you look carefully there, the first coordinates, 0, 8, 6, 7, … are not anything like the second coordinates: 37, 36, 35, 30, … The files are very different, but the models are *at least topologically* the same. They are both caffeine.

#### 2. *Does this structure and/or SMILES string contain this particular substructure?*

Replacing the keyword “SMILES” with “SMARTS” in the above example turns this into a substructure search. Thus, ***LOAD files “$butane” “$hexane”; print {2.1}.find(“SMARTS”, {1.1}, true)*** gives the result *({14:19})*, which turns out to be the full set of six carbon atoms in the hexane model, corresponding to all possible arrangements of SMARTS search CCCC within SMILES string CCCCCC. Adding a third parameter *true* to the find SMARTS function, ***PRINT {2.1}.find(“SMARTS”,{1.1},true)***, gives instead an array of all possible matches. We expect three, because the CCCC could start at the 1st, 2nd, or 3rd atom in the chain, and that is what we get: *[({14:17}),({15:18}),({16:19})].* Note that these three 4-atom sets do not include H atoms.

The use of aromatic directives is particularly useful in a situation where one wishes to compare two versions of a drawn structure. They give us an opportunity to qualify our search: Do the models agree in terms of overall aromaticity? If({*}.find(“SMILES”,“c1(O)ccccc1O”)){…} or, given that that is true, are their Kekulé structures different?

- If (!{*}.find(“SMILES”,“/noaromatic/C1(O)=*CC*=*CC*=*C1(O)”)){…}*

#### 3. *Given two structures, what is their isomeric relationship?*

The directives /nostereo/ and /invertstereo/ can be effectively used to compare two 3D structures, a 3D structure and a reference stereochemical SMILES string, or two stereochemical SMILES strings. The pseudo-code for a full isomeric determination is as follows:

- *If (the molecular weights are different) Return NONE*
- *If (A.matches(B)) Return IDENTICAL*
- *If (!A.matches(/nostereo/B) Return CONSTITUTIONAL ISOMERS*
- *If (A.matches(/invertstereo/B) Return ENANTIOMERS*
- *Return DIASTEREOMERS*

Note that both the /nostereo/ and the /inverstereo/ directives are used to good effect here. In Jmol, this is basically what is carried out behind the scenes [23] in getRelationship(String smiles1, String smiles2). This method is invoked when the Jmol command ***PRINT compare(A, B,“ISOMER”)*** is issued. Here again, ***A*** and ***B*** can be any combination of 3D model atoms and SMILES strings. The importance of the/invertstereo/directive is clear: We do not want to be manually inverting the stereochemistry of SMILES strings. In addition, in this case where ***B*** is a structure and not a SMILES string already, Jmol is internally generating the SMILES string for ***B*** and then prepending these directives en route to its SMILES processor module.

#### 4. *Given two structures from two different sources, how quantitatively similar are they?*

Structures used in Jmol are often derived from a variety of databases, both computational and experimental. The question arises as to how much different such structures are from one another. In this case what is needed is a 1:1 atom mapping between the two structures followed by an alignment. The problem is that the two structure files likely have completely different atom order, and also there could be several suitable mappings. Jmol uses (relatively standard) Jmol SMILES matching to generate this mapping and then uses a quaternion eigenvalue algorithm [24] for the alignment, checking each possibility and looking for the best-fit RMSD. This guarantees that we end up with the very best fit of all possible mappings. If A and B are two 3D structures loaded into Jmol, then their similarity is found by **compare(A,B,”SMILES”, “stddev”)**, where the result is expresses as a standard deviation. The entire calculation is complete in a fraction of a second.

#### 5. *How can I align two 3D models in order to visualize their similarity?*

**Fig. 2:**
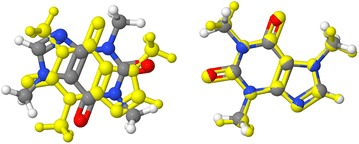
Caffeine from PubChem (*yellow*) and NCI/CADD (*standard colors*) before and after SMILES-based alignment

If we remove that last parameter, the return will be the 4 × 4 rotation–translation matrix describing how to best align the atoms of A onto B. We can effect that overlay of atoms for a visual comparison using the ***rotate selected*** command, as shown in Fig. 2. The following script generates a visual comparison of the caffeine structure found at NCI/CADD with the one at PubChem:

- LOAD files “$caffeine” “:caffeine”; FRAME *
- ***VAR A*** **=** ***{1.1}; B*** **=** ***{2.1}***
- ***VAR m*** **=** ***compare(A, B, “SMILES”)***
- ***SELECT A***
- ***ROTATE selected @m***
- ***COLOR @B yellow***

(Variable ***m*** here is the 4x4 optimal rotation/translation matrix.) In this case we have a very good fit, with RMSD 0.080 Å for all non-hydrogen atoms.

We can also do this alignment using a substructure. So, for example, if we wanted to align these two models specifically using the five-membered ring, we could use a SMARTS search for Cn1cncc1. Substituting above **VAR*m*** **=** ***compare(A, B, “SMARTS”, “Cn1cncc1”)***. Finally, in Jmol there is still a simpler way. The combination of SMARTS- or SMILES-based mapping and quaternion-based alignment can be done in one go using the COMPARE command:

- ***COMPARE {1.1} {2.1} SMILES rotate translate***
- ***COMPARE {1.1} {2.1} SMARTS “Cn1cncc1” rotate translate***

#### 6. *What would I need to do to the given conformation of Structure A to match it conformationally with Structure B? or with some substructure within B?*

**Fig. 3:**
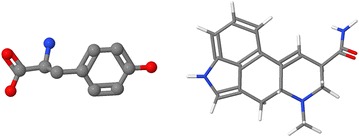
Tyrosine (ball and stick) and lysergamide (wireframe)

**Fig. 4:**
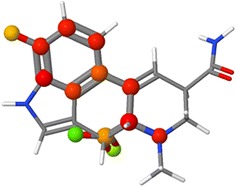
Fully conformationally aligned tyrosine (ball and stick, *colored* by distance to nearest atom of lysergamide) and lysergamide (wireframe)

One very powerful combination of these features also adds dihedral driving—the ability to rotate dihedrals in a way that allows flexible conformational fitting prior to quaternion rotation. In this case, a SMARTS search identifies the key bonds that need to be driven. This is illustrated in Figs. 3 and 4. The following Jmol script loads two models, one of tyrosine and one of lysergamide, displaying them in ball&stick and wireframe, respectively:

- ***LOAD files “$tyrosine” “$lysergamide”***
- ***SELECT 2.1; TRANSLATESELECTED {10 0 0} // just get the two models about 10 ang. apart***
- FRAME *; ZOOMTO 0 {*} 0; // show all models, and zoom into show all of them
- ***SELECT {2.1}; WIREFRAME only // select the second model and make it just thin lines***
- ***DISPLAY remove 1.1 and _H // remove the 1st model’s hydrogen atoms from the display***

The Jmol command ***COMPARE {1.1} {2.1} BONDS “c1ccccc1CCN” rotate translate*** does the final magic. Using a SMARTS search, it matches atoms in the two structures, identifies the associated bonds, calculates all relevant dihedral angles in tyrosine, then rotates all of those dihedrals to positions that match their counterparts in lysergamide. Quaternion-base alignment and animated overlay then transports the conformationally modified tyrosine to its best-fit location within the lysergamide molecule (Fig. 4). A bit of coloring highlights the success of the operation by assigning color in tyrosine (model 1.1) based on distance to the nearest atom in lysergamide (model 2.1):

- ***{1.1}.property_d*** **=** ***{1.1}.distance.min({2.1})***
- ***SELECT{1.1}; COLOR balls property_d***

#### 7. *Given a cyclohexane structure, is it in the chair or boat form? Are substituents axial or equatorial?*

**Fig. 5:**
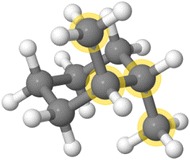
Trans-diaxial conformation selected by Jmol SMARTS selected using ***SELECT on search(“[CH3](.t:*** **-** ***170,*** **-** ***180,170,180)CC[CH3]”)***

The capability to match ranges of values for distances, angles, and dihedrals in Jmol SMARTS allows conformational identification of structures. All that is needed is a careful definition of whatever motif is desired. For example, the various chair conformations of 1,2-dimethylcyclohexane can be distinguished by torsional angles involving ring carbons and the methyl groups (Fig. 5):

cis-1,2: **[CH3](.t:-170,-180,170,180)C1CCC(.t:50,70,-50,-70)CC1[CH3]**
trans-1,2-diequatorial: **[CH3](.t:-170,-180,170,180)C1CCC(.t:-170,-180,170,180)CC1[CH3]**
trans-1,2-diaxial: **[CH3](.t:-170,-180,170,180)CC[CH3]**

In general, we can describe *gauche* as, roughly, *(.t:50,70,-50,-70)***, *eclipsed* as *(.t:-20,20)*** and *anti* as *(.t:170,180,-170,-180)***. Note that in all these cases we are allowing for some nonideality of structures. *Anti* may or may not be 180-degree dihedral. We allow 10 degrees plus-or-minus.

#### 8. *How can I correlate 2D and 3D chemical structures from different sources? For example, how can I correlate a given 2D or 3D structure with a simulated NMR spectrum?*

**Fig. 6:**
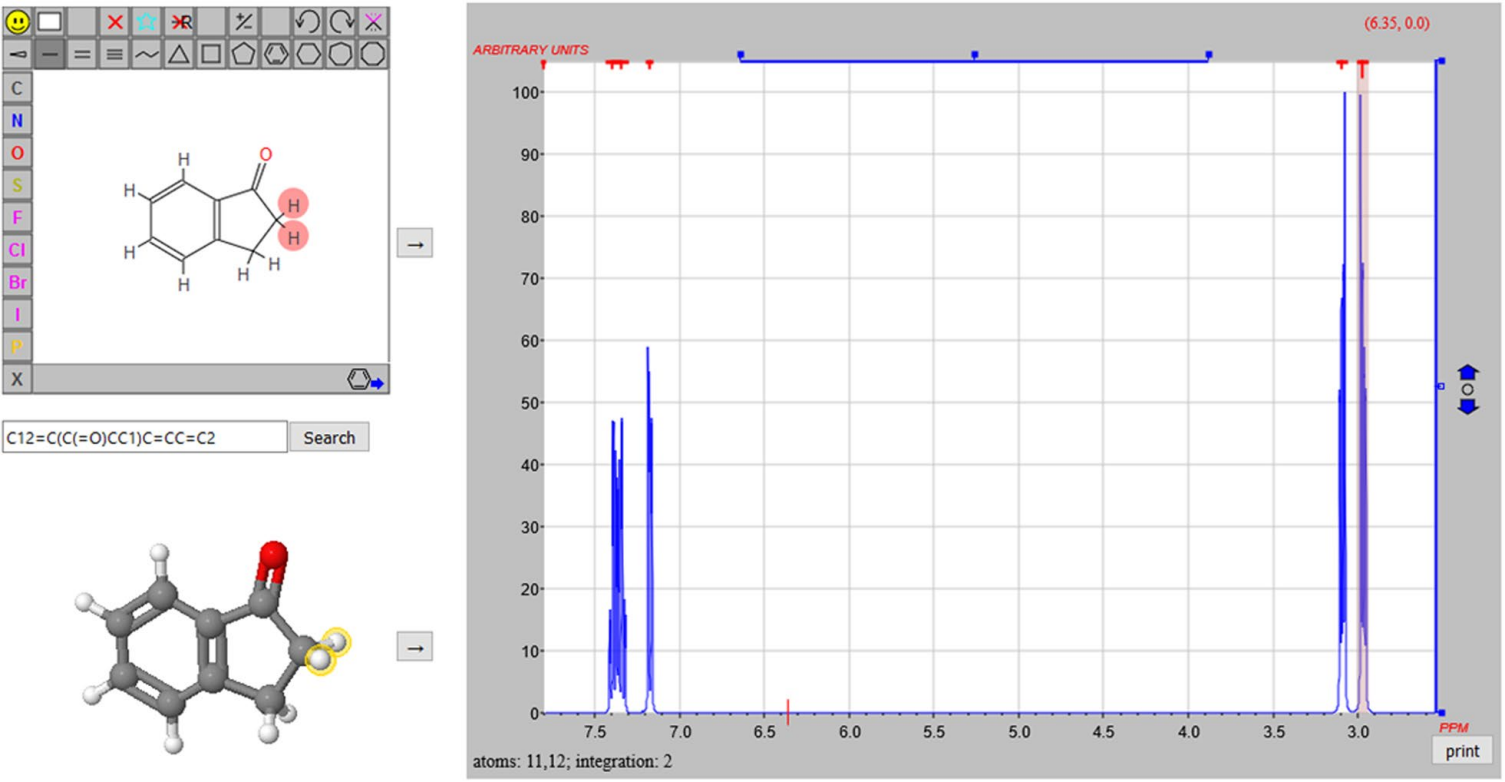
A web application using SMILES to coordinate selection of atoms in 2D and 3D structures, with correlation to simulated 1HNMR spectra

The capability of HTML5 and JavaScript to allow on a single web page a 2D drawing app (JSME), a 3D visualization app (Jmol), and an NMR spectroscopy simulation client (JSpecView [25], a component of Jmol) provides both an opportunity and a challenge. We can, in principle, correlate atoms in the 2D drawing, atoms in the 3D interactive structure, and peaks in the NMR spectrum, thus allowing the user seamless clicking with visual references updating simultaneously in all three apps (Fig. 6) [26]. The challenge is to do the atom–atom mapping necessary to make that work. This is especially challenging because the services that provide the 2D and 3D structures on the page and the 3D structure that is used in the spectral analysis all come from different sources. And to make it even more challenging, an online spectral analysis may return a correlation to a *different* 3D structure than was sent to it. Though “canonical” on their own, these services are anything but canonical as a suite!

The JSmol solution required two atom correlations—from 2D to 3D, and from 3D to 3D—including H atoms, which are not usually part of a SMILES match. A variation of the Jmol compare() function was developed for this purpose: ***atommap*** **=** ***compare({1.1} {2.1} ‘MAP’ ‘H’)***. Here model 1.1 is the structure on the bottom left in Fig. 6; model 2.1 is the model derived from the 2D JSME drawing app above it. “MAP” indicates we want a correlation, and “H” means we want a SMILES all-atom correlation, which includes hydrogen atoms. The variable ***atommap*** is assigned an array of arrays, [[a1, b1], [a2,b2], ….], indicating the exact 1:1 correlation of these two structures in terms of atom indices. The correlation between Jmol and JSpecView in the end was not done using SMILES. Instead, the JSV application matches atoms structures returned by the server by matching individual 3D atom positions. But it would have been possible to use this same compare() function with that comparison as well. Non-canonical SMILES comparison is also being used on this page just to check that the apps are well synchronized and that all models are identical:

- ***jsmeSMILES*** **=** ***jme._applet.smiles();*** (JavaScript)
- ***if(!{1.1}.find(“SMILES”,javascript(jsmeSMILES)))…*** (in JSmol)

Of course, this is all done virtually instantaneously behind the scenes; the page visitor simply sees a well-coordinated application that behaves quite naturally.

## Conclusions

In this article I have presented a set of additions to standard SMILES and SMARTS that allow for powerful applications in 3D structure visualization, comparison, and analysis. Jmol SMILES additions are minimal. Jmol SMARTS atom primitive additions widen the scope of SMARTS searching capability, adding features that are applicable to 3D structures and useful in Jmol, such as allowing Jmol to create atom types for MMFF94 calculations efficiently. Additional atom specifications allow for application-specific atom selection based on criteria not included in any SMARTS specification as well as patterns that are specific to wwPDB-derived models, the ability to specify a variable number of repeating patterns, and the substitution of predefined variables. Non-primitive Jmol SMARTS options include the allowance for subset selection, conformational matching, overall pattern logic, and predefined variables. The result is a rich language for 3D molecular investigation and comparison that greatly expands the usefulness of SMARTS pattern matching.

Additional extensions to Jmol SMILES and Jmol SMARTS that are specific to biopolymers and also extend SMARTS searching to inorganic and periodic crystal structure and to polyhedra analysis are being implemented in Jmol and will be addressed in future communications.

## Supplemental material

Jmol scripts for all example in this article are provided as Additional file 1. All figures in this article are included as PNGJ format files in Additional file 2. These “image + data” files can be drag-dropped or otherwise loaded into Jmol or JSmol to reproduce the 3D model exactly as it appears in the image. Exact scripts used for their creation can be found in Additional file 1.

## Additional files

10.1186/s13321-016-0160-4 Jmol scripts.
10.1186/s13321-016-0160-4 PNGJ image+data files for figures.

## Footnotes

1 The algorithm used by Jmol to identify flat aromatic rings involves the following steps: (1) A set of normals is generated as follows: (a) For each ring atom, construct the normal associated with the plane formed by that ring atom and its two nearest ring-atom neighbors. (b) For each ring atom with a connected atom, construct a unit normal associated with the plane formed by its connecting atom and the two nearest ring-atom neighbors. (c) If this is the first normal, assign vMean to it. (d) If this is not the first normal, check vNorm.dot.vMean. If this value is less than zero, scale vNorm by −1. (e) Add vNorm to vMean. (2) The standard deviation of the dot products of the individual vNorms with the normalized vMean is calculated. (3) The ring is deemed flat if this standard deviation is less than the selected cutoff value, which in Jmol is 0.1 in the presence of /strict/ and 0.01 otherwise.

## References

[1] Weininger D (1988). SMILES, a chemical language and information system. 1. Introduction to methodology and encoding rules. J Chem Inf Comput Sci.

[2] Weininger D, Gasteiger J (2003). SMILES: a language for molecules and reactions. Handbook of chemoinformatics.

[3] Open Smiles. http://www.opensmiles.org/

[4] Daylight Inc. 4. SMARTS—a language for describing molecular patterns. http://www.daylight.com/dayhtml/doc/theory/theory.smarts.html

[5] OpenSMARTS Specification (draft Sept 2012). http://www.moldb.net/opensmarts

[6] Siani MA, Weininger D, Blaney JM (1994). CHUCKLES: a method for representing and searching peptide and peptoid sequences on both monomer and atomic levels. J Chem Inf Comput Sci.

[7] Siani MA, Weininger D, James CA, Blaney JM (1995). CHORTLES: a method for representing oligomeric and template-based mixtures. J Chem Inf Comput Sci.

[8] Drefahl A (2011). CurlySMILES: a chemical language to customize and annotate encodings of molecular and nanodevice structures. J Cheminform.

[9] Ash S, Cline MA, Homer RW, Hurst T, Smith GB (1997). SYBYL Line Notation (SLN): a versatile language for chemical structure representation. J Chem Inf Model.

[10] Homer RW, Swanson J, Jilek RJ, Hurst T, Clark RD (2008). SYBYL Line Notation (SLN): a single notation to represent chemical structures, queries, reactions, and virtual libraries. J Chem Inf Model.

[11] Proschak E, Wegner JK, Schüller A, Schneider G, Fechner U (2007). Molecular Query Language (MQL)—a context-free grammar for substructure matching. J Chem Inf Model.

[12] Yang C, Tarkhov A, Marusczyk J, Bienfait B, Gasteiger J, Kleinoeder T, Magdziarz T, Sacher O, Schwab C, Schwoebel J, Terfloth L, Arvidson K, Richard A, Worth A, Rathman J (2015). New publicly available chemical query language, CSRML, to support chemotype representations for application to data mining and modeling. J Chem Inf Model.

[13] Hanson RM (2010). Jmol—a paradigm shift in crystallographic visualization. J Appl Crystallogr.

[14] Delano W. PyMOL molecular graphics system. https://sourceforge.net/projects/pymol/

[15] Humphrey W, Dalke A, Schulten K (1996). VMD—visual molecular dynamics. J Mol Graph.

[16] Pettersen E, Goddard T, Huang C, Couch G, Greenblatt D, Meng E, Ferrin T (2004). UCSF Chimera—a visualization system for exploratory research and analysis. J Comput Chem.

[17] The PubChem Project. https://pubchem.ncbi.nlm.nih.gov

[18] NCI/CADD Group Chemoinformatics Tools and User Services. https://cactus.nci.nih.gov

[19] Accelrys. CTfile formats. http://download.accelrys.com/freeware/ctfile-formats/ctfile-formats.zip

[20] Halgren TA (1996). Merck molecular force field. I. Basis, form, scope, parameterization, and performance of MMFF94. J Comput Chem.

[21] Hanson RM. MMFF94-smarts.txt. http://sourceforge.net/p/jmol/code/HEAD/tree/trunk/Jmol/src/org/jmol/minimize/forcefield/data/MMFF94-smarts.txt

[22] Bienfait B, Ertl PJSME (2013). a free molecule editor in JavaScript. J Cheminform.

[23] Hanson R. Jmol SmilesMatcher. https://sourceforge.net/p/jmol/code/HEAD/tree/trunk/Jmol/src/org/jmol/smiles/SmilesMatcher.java?format=raw. SmilesMatcher.java method getRelationship(String smiles1, String smiles2)

[24] Horn KP (1987) Closed-form solution of absolute orientation using unit quaternions. J Opt Soc Am A 4:629–642. http://www.opticsinfobase.org/viewmedia.cfm?uri=josaa-4-4-629&seq=0

[25] Hanson RM, Lancashire R (2012) Making the connection between molecular structure and spectroscopy: Jmol, JSpecView, and JCAMP-MOL. Abstracts of Papers of the American Chemical Society 244

[26] Hanson, RM. JSpecView/JSME/nmrdb/NCI-Resolver demo. http://chemapps.stolaf.edu/jmol/jsmol/jsv_predict2.htm

