# Supplementary figures and images for: Jmol SMILES and Jmol SMARTS: specifications and applications
Source: J Cheminform. 2016 Sep 26;8:50. doi: 10.1186/s13321-016-0160-4 (PMC5037863; doi:10.1186/s13321-016-0160-4)

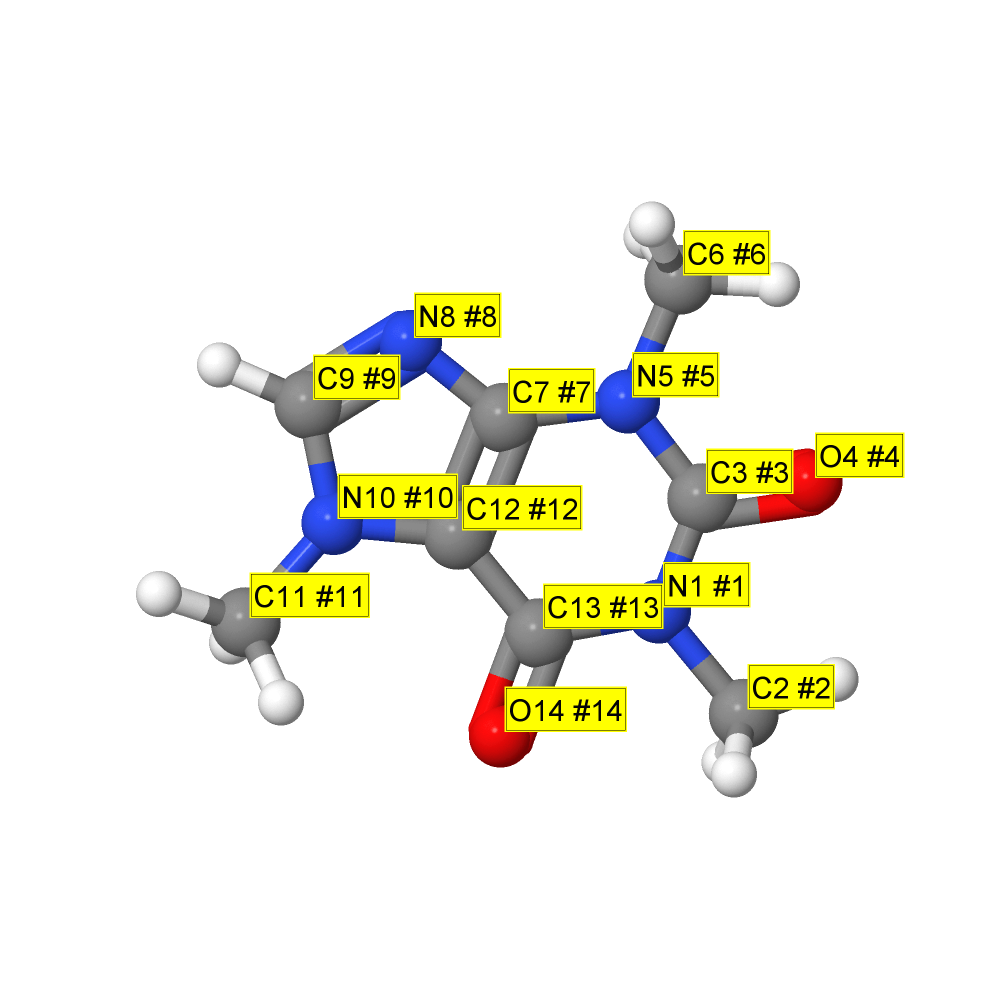

Supplement: Supplementary file 2 — 10.1186/s13321-016-0160-4 PNGJ image+data files for figures. [file 13321_2016_160_MOESM2_ESM.zip › Figure1_large.png]

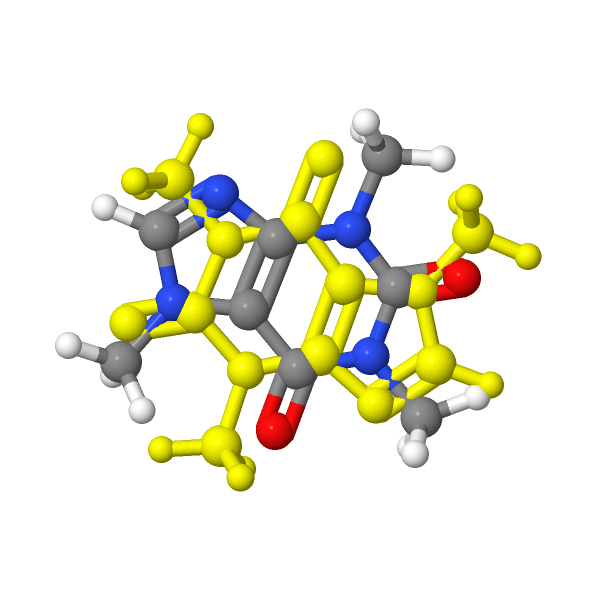

Supplement: Supplementary file 2 — 10.1186/s13321-016-0160-4 PNGJ image+data files for figures. [file 13321_2016_160_MOESM2_ESM.zip › Figure2_largeA.png]

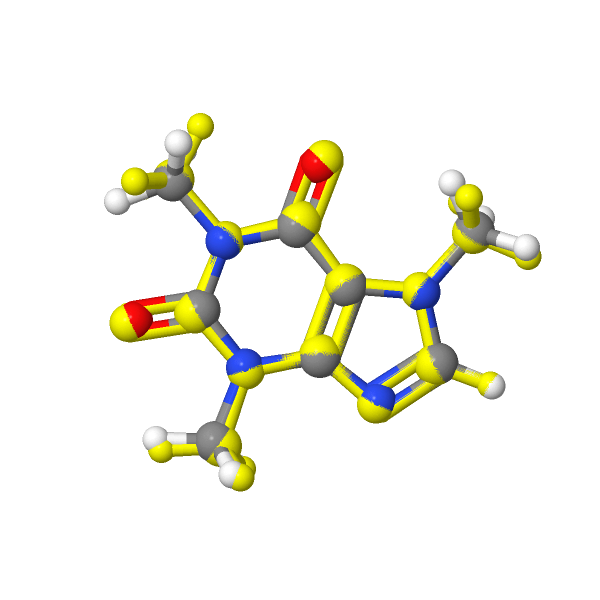

Supplement: Supplementary file 2 — 10.1186/s13321-016-0160-4 PNGJ image+data files for figures. [file 13321_2016_160_MOESM2_ESM.zip › Figure2_largeB.png]

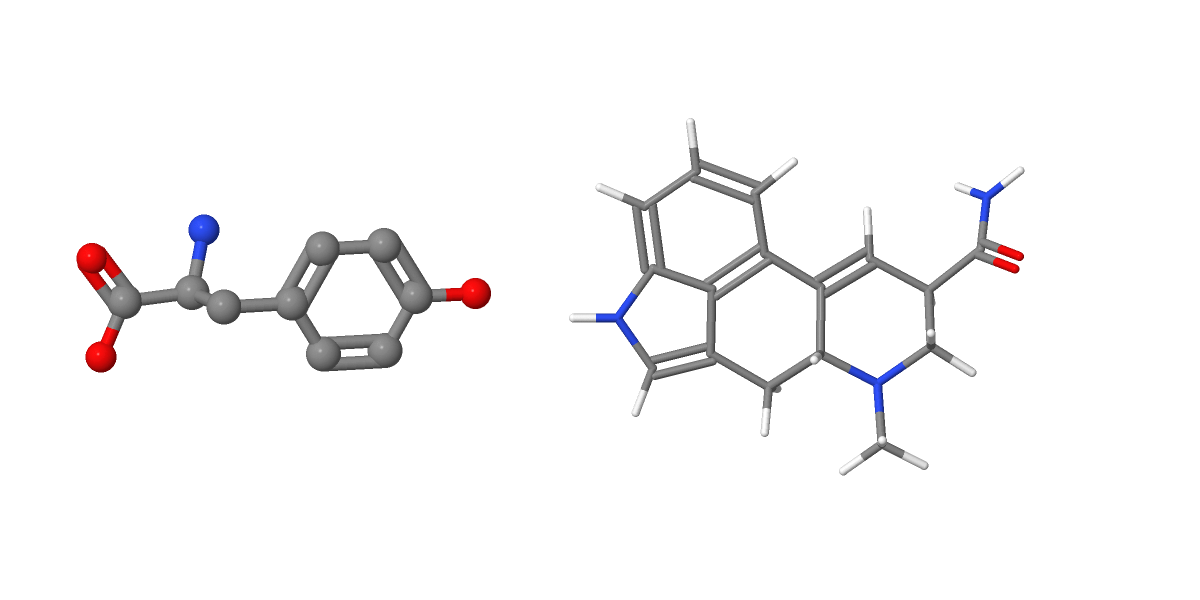

Supplement: Supplementary file 2 — 10.1186/s13321-016-0160-4 PNGJ image+data files for figures. [file 13321_2016_160_MOESM2_ESM.zip › Figure3_large.png]

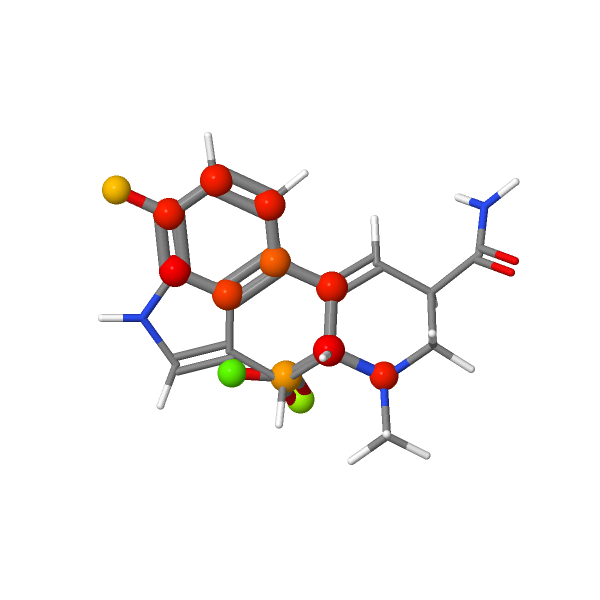

Supplement: Supplementary file 2 — 10.1186/s13321-016-0160-4 PNGJ image+data files for figures. [file 13321_2016_160_MOESM2_ESM.zip › Figure4_large.png]

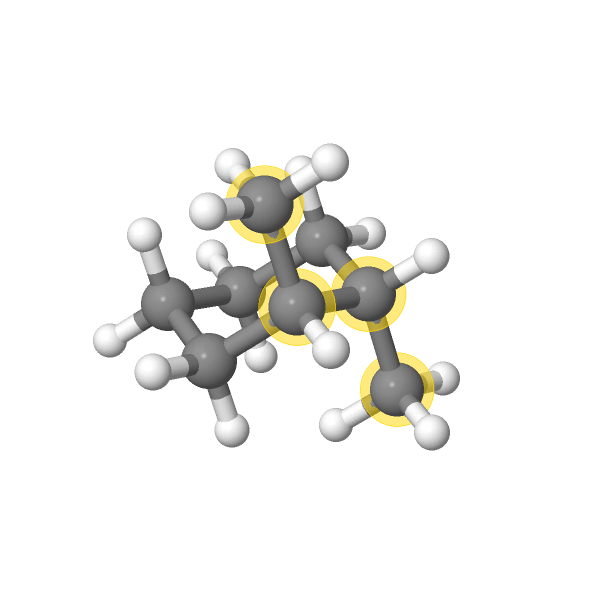

Supplement: Supplementary file 2 — 10.1186/s13321-016-0160-4 PNGJ image+data files for figures. [file 13321_2016_160_MOESM2_ESM.zip › Figure5_large.png]

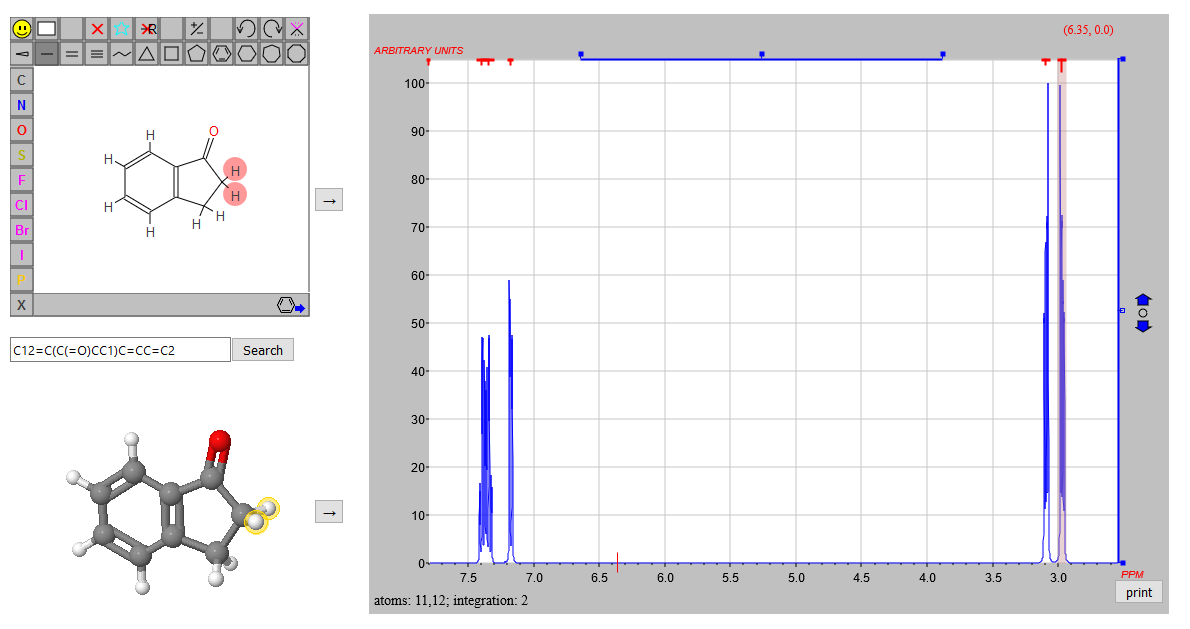

Supplement: Supplementary file 2 — 10.1186/s13321-016-0160-4 PNGJ image+data files for figures. [file 13321_2016_160_MOESM2_ESM.zip › Figure6_large.png]
